# Perceptions of Health-Related Information on Facebook: Cross-Sectional Study Among Vietnamese Youths

**DOI:** 10.2196/ijmr.8072

**Published:** 2017-09-07

**Authors:** Melvyn WB Zhang, Bach Xuan Tran, Huong Thi Le, Hinh Duc Nguyen, Cuong Tat Nguyen, Tho Dinh Tran, Carl A Latkin, Roger CM Ho

**Affiliations:** ^1^ Biomedical Global Institute of Healthcare Research and Technology National University of Singapore Singapore Singapore; ^2^ Institute for Preventive Medicine and Public Health Hanoi Medical University Hanoi Vietnam; ^3^ Bloomberg School of Public Health Johns Hopkins University Baltimore, MD United States; ^4^ Hanoi Medical University Hanoi Vietnam; ^5^ Institute for Global Health Innovations Duy Tan University Da Nang Vietnam; ^6^ Vietnam-Germany Hospital Hanoi Vietnam; ^7^ Yong Loo Lin School of Medicine National University of Singapore Singapore Singapore

**Keywords:** health information, social media, Facebook, beliefs

## Abstract

**Background:**

While health information websites may have previously been the core source of information about health-related conditions on the Internet, social networking sites are increasingly replacing those websites as a source of health-related information. The increasingly popularity of social networking sites among the general population has consequential impact on health policies as well as health-related interventions. To date, there remains a paucity of research conducted in developing countries like Vietnam looking at the influence of social networking sites.

**Objective:**

Our goal is to establish the baseline use of Facebook among Vietnamese youths and establish their perception of the reliability and usefulness of health-related information that they previously encountered while using the social networking site.

**Methods:**

An online cross-sectional study was conducted from August 2015 to October 2015. Respondent-driven sampling (RDS) technique was used in the recruitment of participants. Sociodemographic, health status, behaviors, Facebook use and belief of information on Facebook, and interpersonal influence of social network sites were collected via an online structured questionnaire.

**Results:**

Among 1080 participants, 72.87% (787/1080) reported being interested in health information on Facebook, and 50.74% (548/1080) and 17.50% (189/1080) perceived the information to be reliable and useful, respectively. A total of 10.93% (118/1080) of the participants also reported that they would follow the health advice they obtained from Facebook. Of significance, 7.13% (77/1080) of the participants also reported peer influences on their behavior. Factors that mediate Vietnamese perceptions of the information online include gender, level of perceived stress, age, educational level, and interpersonal influences from Facebook.

**Conclusions:**

Our study is perhaps one of the first conducted in Vietnam that looks at the relationship between health information on Facebook and factors that might influence young Vietnamese perceptions of the information and the consequential use of that information.

## Introduction

Over the past decade, there have been rapid advances in Internet as well as mobile technologies. Such rapid advances in technology have revolutionized health care. Aside from the introduction and implementation of electronic medical records in the health care setting, the availability of the Internet has allowed individuals to seek out information about various medical disorders more readily [1]. It is pertinent to note that the influence of such technological advances is not limited to developed countries. Developing countries such as Vietnam are also becoming increasingly more connected to the Internet [2]. Prior studies conducted have reported that the penetration rate of the Internet in Vietnam has increased approximately threefold since 2005 [2].

More recent research has reported that youths in Vietnam tend to use the Internet, especially using their mobile devices, and those who are younger are also more likely to use social networking sites as well as messaging apps [3].

While technology has transformed how health care professionals function in their day to day activities, it has also affected the general public. There are myriad studies highlighting how technology has empowered individuals and transformed the way they seek out health-related information on the Internet. While health information websites may have previously been the core source of information about health-related conditions on the Internet, social networking sites are increasingly replacing those websites as a source of health-related information. Prior studies have reported there has been a paradigm shift in terms of information-seeking from the health information websites to social media of late [4]. A recent study has looked into how young adults sought information about diabetes and mental health online [5]. Based on the semistructured interviews conducted, it was noted that while young adults do use search engines to look for health-related information, they are increasingly using social media to seek health-related information as well [5]. The increasing popularity of social networking sites among the general population has consequential impact on health policies as well as health-related interventions. Huesch et al [6] have examined how social networking sites such as Facebook could potentially be used for public health interventions. Other research has highlighted the potential utility of social networking sites in reaching out to individuals in the general population who have experienced auditory hallucinations [7]. The research also reported how social networking sites such as Facebook could potentially be used as a form of support and a therapeutic tool for participants who are distressed by their hallucinations [7]. Aside from the use of Facebook for hallucinations, other researchers have proposed that Facebook could also be used to help reduce the incidence of problem drinking among university students through modification of social norms about drinking [8]. Facebook has also been used previously in the analysis of beliefs about common disorders such as attention-deficit hyperactivity disorder [9]. Clearly, there is a huge amount of potential for social networking sites to not only disseminate health-related information but also potentially be used as an intervention.

Granted, prior studies have documented the utility of Facebook as an intervention as well as a resource for health-related information. More recently, a study was conducted to determine the factors associated with how college students seek out health-related information using social networking sites [10]. Another study reported that various sociodemographic variables as well as the sources of the information are influential in participant receptiveness and preferences. Other research has demonstrated how Facebook could help augment tobacco prevention strategies taught in the classroom setting [11]. Notably, that study reported that a good proportion of students posted antitobacco messages on their social media account days after being exposed to tobacco prevention strategies in a classroom setting. Clearly, it is evident that Facebook is being used and perceived as a good tool to communicate health-related information [11].

In a developing country like Vietnam, with the rapid proliferation and increasing affordability of the Internet, it is thus pertinent for us to understand how youths perceive the health-related information on social networking sites. An understanding of this is pertinent prior to the conceptualization and implementation of interventions to deal with public health problems, modeling against research that has been done in the west. To our knowledge, there have not been any prior studies looking into how youths seek out health-related information on social networking sites in Vietnam. Here, thus far, the most common methodology of disseminating health-related information is via school-based campaigns [12].

The aim of this study is to establish the baseline use of Facebook among Vietnamese youths and establish their perception of the reliability and usefulness of health-related information that they previously encountered while using the social networking site. This study also seeks to determine how Vietnamese youths use the information they have obtained from online social networking sites. In addition, this study seeks to determine if there are other variables that might affect youth perceptions and receptiveness, such as their baseline health status. Understanding this information will help guide future public health interventions that use social networking sites as an interventional tool.

## Methods

### Study Setting and Population

In order to achieve the study’s objectives, an online cross-sectional study was conducted from August 2015 to October 2015 in Vietnam. The inclusion criteria for participation in the study include the following: aged 15 to 25 years, currently residing in Vietnam, and having access to an email or social network site account. There are no specific exclusion criteria for this study.

### Respondent-Driven Sampling and Sample Size

For this cross-sectional study, the investigators made use of the respondent-driven sampling (RDS) technique in the recruitment of the participants. The sampling technique is as described elsewhere [13-15]. First, several core participants were recruited from high schools (Hung Yen high school and Phan Boi Chau high school) and universities (Hanoi Medical University and Vietnam National University) in Vietnam. The core participants were carefully selected to reflect the diversity of the Vietnamese population, taking into consideration age, gender, and level of education. Participants in the core groups were required to give their informed consent for the study and were told they would be required to make use of their email or social network site accounts to recruit other students to participate in the study. All participants in the core group were required to complete the Web-based study questionnaire prior to recruiting other participants. The invited participants were provided with the Web link of the survey form for them to complete the same questionnaire. There was no predefined end date of recruitment. The network was allowed to expand until it was deemed to be not able to expand any further. The survey was then deemed complete.

All the participants in the core group as well as the participants whom they invited were included in the final sample. Duplicate participants were identified through email, and amounted to a total of 7 cases, while 3 cases did not meet the inclusion criteria. Participants who did not complete at least 60% of the questions in the survey questionnaire were also excluded from the analysis. The resultant cumulative total sample size amounted to 1080 participants.

### Web Survey Design

The cross-sectional survey was implemented and deployed using Google Forms. All the data acquired from the Web-based study was stored on Google’s Health Insurance Portability and Accountability Act–compliant server. Prior to the commencement of the survey, all participants were provided with information about the study’s purposes and methodology as well as information about the principal and coinvestigators. The questionnaire survey comprised 40 questions, and participants were required to answer 23 questions. The Web survey was piloted among a group of 20 youths of different ages and genders prior to the actual implementation. The participants in the pilot group assisted in the assessment of the usability and reliability of the Web-based survey across a variety of devices and operating system platforms. For the questionnaire, a logic check was implemented in order to ensure that the answers provided corresponded to the theme of the questions.

The Web-based survey included the following questionnaires:

- Baseline demographics questionnaire: Baseline demographic information such as age, gender, educational level, occupational status, marital status, ethnicity, and religion beliefs were acquired from the participants.
- Health-related quality of life (HRQOL): HRQOL was measured by using the EuroQol 5 dimension 5 level (EQ-5D-5L) instrument. The HRQOL questionnaire included 5 domains: mobility, self-care, usual activities, pain/discomfort, and anxiety/depression, with 5 levels of response: no problems, slight problems, moderate problems, severe problems, and extreme problems, giving 3125 health states with respective single indexes. To compute those indexes, the interim scoring for the EQ-5D-5L from the cross-walk value set of Thailand was used due to the unavailability of the Vietnamese population’s reference [16]. Additionally, the EQ-VAS (visual analog scale) assessed the self-rated health of respondents on a 20-cm vertical scale with the endpoint range from 0 to 100 points, labeled “the best health you can imagine” and “the worst health you can imagine.” The validated Vietnamese version of the EQ-5D-5L has been used elsewhere [13,14,17-22].
- Stress measurement: The short-form Perceived Stress Scale (PSS) was used to measure the stress of participants in the last 30 days. This instrument included 4 items with a 5-point scale: never (0), almost never (1), sometimes (2), fairly often (3), very often (4). Two items were negatively coded (‘‘In the last month, how often have you felt that you were unable to control the important things in your life?’’ and ‘‘In the last month, how often have you felt difficulties were piling up so high that you could not overcome them?’’) and 2 items are positively coded (‘‘In the last month, how often have you felt confident about your ability to handle your personal problems?’’ and ‘‘How often have you felt that things were going your way?’’). In scoring the stress measure, positive items are reverse scored, and then all items are summed (scores ranging from 0 to 16 for the 4-item scale). Higher scores indicate greater stress [23].
- Risk behaviors: Questions were asked as to whether participants have had other risk behaviors such as smoking tobacco products or use of alcohol. To quantify the severity of the alcohol use disorder, we used the Alcohol Use Disorders Identification Test–Consumption (AUDIT-C) instrument, which is a short version of the Alcohol Use Disorders Identification Test. This tool consisted of 3 questions with scoring from 0 to 12, with a higher score indicative of a higher risk of alcohol dependence. If male respondents scored ≥4 or female respondents scored ≥3, they were determined as AUDIT+ [21,24-30].
- Facebook use and belief of information on Facebook: We asked questions relating to the amount of time individuals spent using Facebook on a daily basis, as well as their primary activity on Facebook. In addition, we asked how participants usually obtained information about medical conditions and health-related conditions. Subsequently, participants were asked whether they had any interest in the health information on Facebook, and they were tasked to estimate the amount of time they spent reading health-related information on Facebook. Participants were asked to rate the perceived level of reliability of the health information, as well as the perceived level of usefulness. Participants were also asked how often they had shared health-related information using the social networking site.
- Interpersonal influence of social network sites: Questions were asked to investigate online peer influence. These included talking with new friends on the Internet, going to places introduced by online friends, effect of online friends on behavior and lifestyle, and trying to do something introduced by online friends.

### Statistical Analysis

Chi-square, *t* test, and analysis of variance were used to explore the differences among characteristics. Multivariate logistic regressions were employed to identify the associated factors. In this study, we applied a stepwise forward model strategy that uses a log-likelihood ratio test at a *P* value of .10 to select variables for the reduced models [31]. A *P* value of less than .05 was set as the level of statistical significance.

### Ethical Approval

The Institutional Review Board of the Vietnam Authority of HIV/AIDS Control provided the ethical approval for this study. All participants were asked to provide an electronic informed consent. Participants were briefed and informed that they could withdraw anytime they wanted. Information obtained from the participants was coded and kept confidential.

## Results

A total of 1080 participants took part in the questionnaire survey, out of which 41.94% (453/1080) were males; 54.26% (586/1080) of the individuals were in the 18 to 22 year age group. The vast majority (849/1080, 78.61%) were undergraduate students, 95.83% (1035/1080) were of Kinh ethnicity, and 72.59% (784/1080) were single. A total of 47.59% (514/1080) of the participants resided in rented accommodations. A total of 9.63% (104/1080) of the participants reported having had acute illnesses in the preceding month, and 18.88% (204/1080) of the participants reported that they have had chronic illnesses in the last 3 months. Among those sampled, 9.26% (100/1080) were overweight. In terms of their HRQOL, the vast majority of the participants reported having experienced mood symptoms (806/1080, 74.63%) as well as pain-related symptoms (548/1080, 50.74%). About 10.74% (116/1080) of the participants were currently smoking, and twice that (235/1080, 21.76%) were using alcohol. A total of 5.19% (56/1080) of the participants had also been smoking shisha. The mean scores for all participants on the EQ-5D and EQ-5D VAS were 0.73 and 80.59, respectively. The mean score for all participants on the PSS was 6.58.

Table 1 provides an overview of the baseline use of Facebook by the participants as well as their perceptions about health-related information on Facebook. The average amount of time participants spent on Facebook was 3.05 hours. The mean hours for females and males were 2.98 and 3.14 hours, respectively, and there were no significance differences between the means. The vast majority of the participants (713/1080, 66.02%) used Facebook primarily to keep themselves updated with the latest news. For most participants, friends are regarded as the primary source of their health information. At least 72.9% (787/1080) of the participants reported that they were interested in the health information shared on Facebook. A total of 65.74% (710/1080) of the participants spent less than 30% of the total time they spent on Facebook engaging in and reading health-related information.

**Table 1:** Use of Facebook for seeking health information among respondents.

Characteristics		Female		Male		Total		*P* value
**Primary purpose for using Facebook, n (%)**								
	Chatting with friends	143 (22.77)	144 (31.86)		287 (26.57)		.011	
	Update news	436 (69.43)	277 (61.28)		713 (66.02)			
	Counseling	6 (0.96)	4 (0.88)		10 (0.93)			
	Playing games	43 (6.85)	27 (5.97)		70 (6.48)			
**Health information sources, n (%)**								
	Friends	267 (42.52)	239 (52.88)		506 (46.85)		.006	
	Health page/groups	220 (35.03)	130 (28.76)		350 (32.41)			
	Talk with consultants	2 (0.32)	0 (0)		2 (0.19)			
	Others	139 (22.13)	83 (18.36)		222 (20.56)			
**Interest in health information shared on Facebook, n (%)**								
	Highly interested	149 (23.73)	83 (18.36)		232 (21.48)		<.001	
	Interested	346 (55.10)	209 (46.24)		555 (51.39)			
	Low interest or not interested	133 (21.18)	160 (35.40)		293 (27.13)			
**Time spent reading health information on Facebook, n (%)**								
	<10%	164 (26.11)	178 (39.38)		342 (31.67)		<.001	
	10%-<30%	224 (35.67)	144 (31.86)		368 (34.07)			
	30%-50%	118 (18.79)	64 (14.16)		182 (16.85)			
	50%-70%	62 (9.87)	25 (5.53)		87 (8.06)			
	>70%	6 (0.96)	4 (0.88)		10 (0.93)			
	Unknown	54 (8.60)	37 (8.19)		91 (8.43)			
Time spent using Facebook per day (hours), mean (SD)		2.98 (2.562)		3.14 (2.901)		3.05 (2.713)		.351

There were, however, statistically significant differences between the genders in terms of their primary purposes of using Facebook (*P*=.011), their health information sources (*P*=.006), their level of interest in health information (*P*<.001), and the amount of time they spent reading health-related information (*P*<.001).

Table 2 provides a detailed analysis of the participant perceptions of the usefulness and reliability of health-related information on Facebook. A total of 50.65% (547/1080) of the participants reported that they felt that the information was moderately reliable, and 17.50% (189/1080) reported that the information was highly reliable. Only 4.91% (53/1080) of the participants perceived the information to be useful. The vast majority of the participants (629/1080, 58.24%) felt that the information was neither useful nor useless. Only 31.20% (337/1080) of the sampled participants reported that they would not share the health information on Facebook. A cumulative total of 10.93% (118/1080) of participants reported that they would follow the health advisory shared on Facebook. There are significant differences between the genders in all the domains discussed.

Table 3 highlights the interpersonal influence of Facebook and social media sites on respondents. Among the participants, 8.17% (79/967) reported that social media encourages them to either talk to or meet new friends online. In addition, 7.07% (68/962) reported that their behaviors, lifestyle, and perceptions are highly affected by others whom they have met online. Of significance, approximately 13.40% (129/963) of participants reported that they ventured to a new place that was introduced by their online friends. In addition, 8.58% (83/967) of participants also reported that they often would try out new activities introduced by their online peers. There was no noted significant difference among the genders and the interpersonal influence, as the *P* values obtained were all not significant.

**Table 2:** The belief of respondents in health information on Facebook.

Characteristics		Female n (%)	Male n (%)	Total n (%)	*P* value
**Reliability of health information shared on Facebook**					
	Low	134 (21.34)	160 (35.40)	294 (27.22)	<.001
	Moderate	364 (57.96)	183 (40.49)	547 (50.65)	
	High	106 (16.88)	83 (18.36)	189 (17.50)	
	Unknown	24 (3.82)	26 (5.75)	50 (4.63)	
**Usefulness of health information shared on Facebook**					
	Not useful	242 (38.54)	156 (34.51)	398 (36.85)	<.001
	Normal	370 (58.92)	259 (57.30)	629 (58.24)	
	Useful	16 (2.55)	37 (8.19)	53 (4.91)	
**Frequency of sharing health information on Facebook**					
	Always	23 (3.66)	34 (7.52)	57 (5.28)	.002
	Often	35 (5.57)	30 (6.64)	65 (6.02)	
	Occasionally	388 (61.78)	233 (51.55)	621 (57.50)	
	Never	182 (28.98)	155 (34.29)	337 (31.20)	
**Practice health information on Facebook**					
	Always	18 (2.87)	20 (4.42)	38 (3.52)	<.001
	Often	49 (7.80)	31 (6.86)	80 (7.41)	
	Occasionally	508 (80.89)	320 (70.80)	828 (76.67)	
	Never	53 (8.44)	81 (17.92)	134 (12.41)	

**Table 3:** Interpersonal influence on Internet among respondents.

Characteristics		Female n (%)	Male n (%)	Total n (%)	*P* value
**Talk and meet new online friends**					
	Often	30 (5.39)	49 (11.95)	79 (8.17)	<.001
	Frequently	101 (18.13)	140 (34.15)	241 (24.92)	
	Rarely or never	426 (76.48)	221 (53.90)	647 (66.91)	
**Effects of online relationships on behaviors, lifestyle, and perceptions**					
	High influence	36 (6.46)	32 (7.90)	68 (7.07)	.466
	Normal influence	138 (24.78)	89 (21.98)	227 (23.60)	
	Low influence or no influence	383 (68.76)	284 (70.12)	667 (69.33)	
**Go to places introduced by online friends**					
	Often	79 (14.21)	50 (12.29)	129 (13.40)	.685
	Frequently	294 (52.88)	221 (54.30)	515 (53.48)	
	Rarely or never	183 (32.91)	136 (33.42)	319 (33.13)	
**Try to do something introduced by online friends**					
	Often	47 (8.44)	36 (8.78)	83 (8.58)	.671
	Frequently	274 (49.19)	212 (51.71)	486 (50.26)	
	Rarely or never	236 (42.37)	162 (39.51)	398 (41.16)	

Multimedia Appendix 1 provides an overview of the multivariate regression analysis to determine factors that are associated with health-related information-seeking using Facebook. Males (odds ratio [OR] 0.42, 95% CI 0.31-0.58) are less likely to be concerned about health-related information on Facebook. Those participants who have reported increased levels of perceived stress, as determined by their scores on the PSS, are also less likely to be interested in and concerned about health information (OR 0.89, 95% CI 0.83-0.95). Factors that mediate whether participants believe the health information they have acquired from Facebook are affected by sociodemographic variables such as age and education. Participants who are older are less likely to believe in the health information provided on Facebook (OR 0.87, 95% CI 0.80-0.95). Participants with vocational training education status are more likely to belief in the health information on Facebook as compared to those with university or postgraduate education. The analysis of interpersonal influences was conducted as we need to determine whether it is one of the associated factors that might mediate the sharing of health-related information by participants. Participants who tend to make use of social media like Facebook to form new relationships (OR 2.65, 95% CI 1.26-5.56) and participants who are easily influenced by their online peers (OR 2.20, 95% CI 1.05-4.60) are more likely to share health-related information using their social networking accounts. With regard to whether participants would adhere to the health information provided on the social network, being of male gender has been found to be negatively associated with adherence (OR 0.84, 95% CI 0.76-0.93). However, interpersonal factors like using the social network to find new friends and following the advice of online peers are associated with a heightened incidence of adhering to the health information provided. Of significance, health information is commonly perceived to be useful among those individuals who are overweight.

## Discussion

### Principal Findings

This is one of the first studies conducted in Vietnam that looks into the relationship between health information on Facebook and associated factors that might influence Vietnamese perceptions of the information and their use of the information that they have obtained from social networking sites. In our study, a total of 1080 participants took part and the vast majority were undergraduate students. A total of 18.88% (204/1080) of the participants reported that they have had health issues over the past 3 months, 9.26% (100/1080) of them were overweight, and a significant proportion have had pain or mood-related symptoms. Among the participants, 72.90% (787/1080) of them reported that they were interested in the health information on Facebook, and 50.65% (547/1080) and 17.50% (189/1080) perceived the information to be reliable and useful, respectively. A total of 10.93% (108/1080) of the participants also reported that they would follow the health advice they obtained from Facebook. Of significance, 7.07% (68/1080) of the participants also reported peer influences on their behaviors. Factors that mediate Vietnamese perceptions of the information online include gender, level of perceived stress, age, educational level, and interpersonal influences of Facebook. These factors moderated and affected how Vietnamese perceive the usefulness and reliability of the health information, as well as the likelihood that they would share the information with their peers.

There have been prior studies conducted in China looking at the equivalents of social networking sites like Facebook and determining factors that might affect how their cohort used social networks [32]. One study reported that Internet self-efficacy is a mediating factor influencing use [32]. Other studies have looked into specific ethnicity and how ethnicity in itself might be a mediating factor affecting use [33]. Lee [33] reported that as many as 98% of African American college students had a Facebook account and were actively using Facebook. In addition, younger users reported spending more time online. A study also reported how cultural factors could influence use [33]. Cultural factors do have a significant influence on the motivation to use Facebook as well as the amount of time devoted to using it. Underwood et al [34] reported that there are fundamentally 3 different types of users—broadcasters, communicators, and high interactors—and the use of Facebook differs among these groups. Other comparative studies have reported on how women from different cultures use Facebook differently [35]. For example, Americans tend to seek out Facebook for entertainment, whereas Koreans tend to seek social support and information online [34-36]. These previous studies highlight the prevalent usage of Facebook across various countries and that the usage might be mediated and influenced highly by cultural variable. Our study has reported that among our sampled cohort of Vietnamese, a good number of individuals were using Facebook and that the mean duration of use was 3.05 hours. In our study, we found that the vast majority of the participants have used Facebook to keep themselves updated about the latest news. The findings from our study are much in line with that of Kim et al [36], and this was expected as ours is an Asian cohort.

Our study, which looks into the acceptability and receptiveness of youths toward health information available on Facebook, is crucial, as a prior study has examined how social media could be used by community-based organizations who are conducting health promotion [37]. That study has examined social networking sites such as Facebook, Twitter, and YouTube and found that these social media networks helped in the dissemination of health information to participants [37]. Our study finds that Facebook could potentially be a good medium for the dissemination of health-related information, given that a good number of our sampled participants believe in the reliability of the information and are keen to share the information.

In our study, we found that males are less likely comparatively to be concerned about health-related information on Facebook. The fact that males are less likely to be concerned about health-related information might be accounted for by gender differences in the usage of Facebook. Raacke and Bonds-Raccke [38] reported that males tend to have a larger social network on Facebook and tend to make use of Facebook for dating-related purposes. In addition, we found that educational level would mediate whether participants believe the health-related information they have obtained online. Of significance, those with only a vocational level of education are more likely to believe the information online as compared to those with higher levels of education. We postulate that this might be related to how participants process the information by determining the accuracy and source of the information. Those with higher levels of education are more likely to consider the accuracy as well as the original source of the health-related information.

In addition, in our study we have determined that Facebook is associated with significant interpersonal influence. A prior study by Hormes [39] has suggested that the excessive use of Facebook is similar to that of a behavioral form of addiction and demonstrated that those who met the criteria for disordered social networking usage were more likely to use alcohol. While Hormes’ study demonstrated the negative impact of Facebook, it has demonstrated that Facebook could potentially shape one’s perceptions. In our study, it is likely that Facebook might have caused changes in perceptions toward health-related information, whereas in Hormes’ study, Facebook normalized alcohol use and provided opportunities for increased use of alcohol. While there remains to date limited literature on interpersonal influences of Facebook, a previous study examining Internet addiction has demonstrated that those with Internet addiction are more susceptible to interpersonal influences due to their reduced cognitive abilities [15]. We postulate that this might be similar for Facebook users, especially for those who are using Facebook excessively.

There are several clinical implications that arise from our study. Given the results, clinicians should be aware that youths and young adults are increasingly exposed to health-related information on social media. Hence, there is a good potential for the use of social media to augment the conventional mechanism of delivery of health-related information. Clinicians should know that information gotten from Facebook might not be entirely factually correct and should provide evidence-based information and recommendations about the various diseases to their patients. It is important to tale gender and educational influences into consideration and for there to be tailored and customizable messages. Clinicians should recognize the role of social media in their outreach efforts because we have found in this study that social media has interpersonal influences and messages that are disseminated could have potentially positive or negative outcomes. Hence, in using Facebook as a medium for health information dissemination, there needs to be some moderation of the content that is posted online. There has been recent research demonstrating how social media use could help in public health campaigning, especially in a low-resource setting [40]. The authors have reported how effective such media are, given that they have inherent potential for rapid transfer of information to the masses [40].

### Strengths and Limitations

Our study aimed to determine the use rates of social networking sites among young Vietnamese. Our study is perhaps one of the first studies examining the rates of social media use to be conducted in a developing country like Vietnam. We recruited a sizeable number of participants who, at the baseline, had knowledge of how to use the Internet (because they were required to make use of the Internet to take part in our survey). In addition, we were able to determine their perceptions and beliefs about health-related information shared on social networking sites as well as the interpersonal influences of social networking sites. We identified their baseline health status and other demographic variables and the influence of these variables on their perceptions of the health-related information shared online. Despite the clear strengths of our study, we do acknowledge several inherent limitations. Our sampled cohort might not be entirely representative of the general Vietnamese population. However, Internet-based sampling is the most effective low-cost way to reach out to individuals in a developing country like Vietnam. Also, our sampling is dependent on RDS and hence our participants needed to have social networking accounts to recruit other participants. We have only examined individual perceptions toward health-related information in general. Ideally, it would be of importance to determine their perceptions toward particular health-related information such as particular diseases, and this will help guide our future interventions.

### Conclusion

This study is perhaps one of the pioneering studies conducted in Vietnam looking at the relationship between health information on Facebook and factors that might influence young Vietnamese perceptions of the information and the consequential use of that information. Clearly, from our study, a good proportion of young Vietnamese use social media to check out health-related information and share this information with their peers. Factors that mediate Vietnamese perceptions of the information online include gender, level of perceived stress, age, educational level, and interpersonal influences of Facebook. The above findings have clinical implications for clinicians who might wish to consider social sites for health-related interventions.

## Appendix

Multimedia Appendix 1 Associated factors with health information seeking behaviors and belief on Facebook among respondents.

## Abbreviations

AUDIT-C: Alcohol Use Disorders Identification Test–Consumption
EQ-5D-5L: EuroQol 5 dimension 5 level questionnaire
HRQOL: health-related quality of life
OR: odds ratio
PSS: Perceived Stress Scale
RDS: respondent-driven sampling
VAS: visual analog scale
