# Supplementary material for: Perceptions of Health-Related Information on Facebook: Cross-Sectional Study Among Vietnamese Youths
Source: Interact J Med Res. 2017 Sep 7;6(2):e16. doi: 10.2196/ijmr.8072 (PMC5608988; doi:10.2196/ijmr.8072)
Supplement: Multimedia Appendix 1 [file ijmr_v6i2e16_app1.pdf]

Multimedia Appendix 1: Associated factors with health information seeking behaviors and belief on Facebook among respondents

Factors	Concern			Belief			Sharing			Practice			Useful		
	OR	95%CI		OR	95%CI		OR	95%CI		OR	95%CI		OR	95%CI	
**Gender (Male vs Female)**	0.42*	0.31	0.58							0.84*	0.76	0.93			
**Age**				0.87*	0.80	0.95									
**Education (vs ≤ High school)**															
Vocational training	4.43	0.96	20.39	6.21*	1.55	24.84				3.96*	1.28	12.24	3.39	1.23	9.28
College	1.66	0.84	3.28	4.59*	1.54	13.71							1.84	1.05	3.23
University				2.56	0.97	6.75									
Graduate				2.48	0.74	8.34									
**Living location (vs Rent hostel)**															
living with family							1.66*	1.03	2.68	2.19*	1.32	3.65			
Living with relatives	0.63	0.37	1.08							1.85	0.80	4.30			
Others							5.56*	1.41	21.87						
**Religion (Cult of Ancestor vs Other)**	1.85*	1.22	2.81	1.84*	1.05	3.20									
**Marital status (Single vs Living with spouse/partner)**							0.47*	0.29	0.76	0.66	0.39	1.14			
**Having acute disease in the last 4 weeks (Yes vs No)**															
**Having chronic disease in the last 3 months (Yes vs No)**	1.34	0.90	1.99							0.44	0.13	1.46	1.28	0.89	1.82
**Overweight/obesity (Yes vs No)**													0.36*	0.20	0.65
**Having usual activities problem (Yes vs No)**													1.53*	1.10	2.14
**Pain/discomfort (Yes vs No)**							1.59	0.89	2.84						
**Perceived stress score**	0.89*	0.83	0.95	0.94	0.87	1.02	0.90	0.80	1.00				0.94	0.88	1.00
**EQ-VAS**													0.99	0.98	1.00
**EQ-index**							0.27	0.06	1.22						
**Current smokers (Yes vs No)**	1.41	0.84	2.39	1.63	0.97	2.75									
**Shisha smokers (Yes vs No)**	0.54	0.27	1.09										0.48	0.23	1.02
**Talk and meet new online friends (vs Rarely/Never)**															
Sometimes							2.66*	1.59	4.46	1.54	0.89	2.69	1.34	0.97	1.85
≥ Often							2.65*	1.26	5.56	2.59*	1.24	5.42	1.72*	1.01	2.92
**Effects of online relationships on behaviors, lifestyle and perception (vs Low/No influence)**															
High				1.54	0.86	2.74	2.20*	1.05	4.60	2.37*	1.16	4.84			
**Go to place introduced by online friends (vs Rarely/Never)**															
Sometimes	1.72*	1.23	2.41				0.67	0.42	1.08						
≥ Often	2.17*	1.29	3.63							1.41	0.75	2.67	2.02*	1.35	3.03
**Try to do anything introduced by online friends (vs Rarely/Never)**															
Sometimes	1.49*	1.09	2.04							1.74	0.99	3.04			
≥ Often										1.84	0.79	4.26			

*p<0.05
